# *Pinellia ternata*, *Citrus reticulata*, and Their Combinational Prescription Inhibit Eosinophil Infiltration and Airway Hyperresponsiveness by Suppressing CCR3+ and Th2 Cytokines Production in the Ovalbumin-Induced Asthma Model

**DOI:** 10.1155/2009/413270

**Published:** 2009-08-02

**Authors:** In-Soo Ok, Seung-Hyung Kim, Bok-Kyu Kim, Jang-Cheon Lee, Young-Cheol Lee

**Affiliations:** ^1^Department of Herbology, College of Oriental Medicine, Sangji University, Wonju 220-702, South Korea; ^2^Institute of Traditional Medicine and Bioscience, Daejeon University, Daejeon 300-716, South Korea; ^3^Division of Pharmacology and Basic Oriental Medicine, School of Oriental Medicine, Pusan National University, Pusan 609-735, South Korea

## Abstract

*Background and Objective*. This study was aimed to analyse the curative effects of *Pinellia ternata*, *Citrus reticulata*, and their combination on airway hyperresponsiveness (AHR) to inhaled methacholine, pulmonary eosinophilic infiltration, Th2 cytokine production, and IgE and histamine production in a murine model of asthma. *Methods*. For this purpose, BALB/c mice were systemically sensitized to ovalbumin (OVA) followed intratracheally, intraperitoneally, and by aerosol allergen challenges for 12 weeks. We examined the development of pulmonary eosinophilic accumulation, control of Th2 cytokine, immunoglobulin E (IgE), and histamine productions in a murine model of asthma. *Results*. Our data suggest that the therapeutic mechanism by which *Pinellia ternata*, *Citrus reticulata*, and their combinational prescription effectively treats asthma is based on reductions of eosinophil infiltration, eotaxin receptor (CCR3), histamine, OVA-specific IgE productions in serum, and Th2 cytokines (IL-5, IL-13) by marked reductions of IL-5 and IL-13 mRNA expression in lung tissue. *Conclusions*. These findings provide evidence that *Pinellia ternata*, *Citrus reticulata*, and their combination play a regulatory role in allergic inflammation and offer therapeutic approaches as novel CCR3 antagonists for treatment asthma. However, it is not clear whether pharmacological activities of prescription composed of two herbs are potentiated due to synergistic effect or additive effect.

## 1. Introduction

Asthma is a chronic inflammatory disease of the airway, which is characterized by the presence of increased numbers of T-helper 2 (Th2) lymphocytes, eosinophils [1], and airway inflammation [2]. It is accompanied by high serum levels of immunoglobulin E (IgE), as well as by intrapulmonary production of interleukin-4 (IL-4), IL-5, and IL-13 by allergen-specific Th2 cells [3]. Airway inflammation is associated with the infiltration of eosinophils, neutrophils, and T and B lymphocytes into airway and lung tissues. 

 In most asthma phenotypes, there are increases in eosinophils in the tissues, blood and bronchoalveolar lavage fluid. Moreover, Th2 cells and their secreted products initiate airway inflammation that begins with the infiltration of eosinophils and other inflammatory cells. The recruited eosinophils produce IL-3, IL-5, IL-6, IL-13, and so forth. 

 Th2 cytokines play an important role in the lungs of asthmatic subjects, particularly because IL-4 and IL-13 enhance immunoglobulin E (IgE) production, IL-5 enhances eosinophil accumulation, and IL-13 directly enhance mucus hypersecretion and airway hyperreactivity (AHR) [4, 5]. 

*Pinellia ternata* (PT), *Citrus reticulata* (CR), and their combinational prescription (composed of PT, CR by a ratio of 50 to 50 based on Korean traditional medicinal formulae) are medicinal herbs and prescription (Leejintang) that have been used for treating airway inflammatory deseases (including asthma) in Korea [6]. PT has the actions of relieving cough, antiemetic, antineoplasm, antifertilization. 

 Previous studies reported that guanosine, tyrosine, and phenylalanine were contained in the tuber of PT [7]. Recently, cytidine, adenosine, tryptophan, uridine, and adenine are reported to be contained for the first time in the tuber of PT by Chen et al. [8]. 

 Pinellic acid (9S, 12S,13S-trihydroxy-10E-octadecenoic acid) has been identified as an orally active adjuvant substance from the tuber of PT [9]. In addition, pinellic acid modulate the production of antigen-specific antibodies in the respiratory tracts and Oral administration of pinellic acid (50 *μ*g/kg/day) also reduced the OVA-specific IgE antibody titer in BAL fluids from the sensitized mouse [10]. 

 CR is consisted of component such as flavonoids, limonoid, polyphenols. Among those, flavonoids affected the inflammation response at human monocytes and citrus nobiletin had inhibitory effect on the reduction of phorbol ester-induced skin inflammation [11–13]. Jung et al. reported that CR-inhibited expression of iNOS and NF-kB activation in LPS-activated RAW 264.7 macrophage cells [14]. Hesperidin [15] of which exhibit antiinflammatory effects, are the active ingredients in *Citrus* flavonoid*.*

 However, there have been no reports of the antiasthmatic and antiinflammatory activities of PT, CR and their combinational prescription in vivo. The aim of this study was to evaluate the ability of them to control Th2-type cytokines, eosinophil infiltration and other factors. Our findings provide evidence that PT, CR and their combinational prescription play a regulatory role in allergic inflammation and offer therapeutic approaches as novel CCR3 antagonists for treatment asthma.

## 2. Materials and Methods

### 2.1. Plant Material and Preparation of Extracts

The tuber of PT, CR and their combinational prescription were purchased from Saerom company (Ansung, South Korea) in February, 2006. The plant was identified by Professor Young-Cheol Lee, College of Oriental Medicine, Sangji University in Wonju, South Korea, and a voucher specimens (PT, CR) are deposited in our laboratory (Department of Herbology, College of Oriental Medicine, Sangji University Wonju 220–702, South Korea). Plant materials (PT, 200 g; CR, 200 g; prescription, 200 g) was extracted three times with 1.5 liter of distilled water at 100°C for 2 hours separately. Then, the extract was filtered and evaporated on a rotatory evaporator (Rotary evaporator, BUCHI B-480, Switzerland) and finally dried by a freeze drier (Freeze dryer, EYELA FDU-540, Japan) to yield the extract PT (20 g), CR (15 g) and their combinational prescription (18 g).The yield (w/w) of the extract was about 10% (PT), 7.5% (CR) and 9% (PT+CR).

### 2.2. Animals

Seven to eight-week-old female Balb/c mice were obtained from Daehan Biolink Co. LTD. (Eumsung, South Korea). All animal procedures were conducted in accordance with the guidelines of the Institutional Animal Care and Use Committee of the South Korea Research Institute of Bioscience and Biotechnology (Daejeon, South Korea).

### 2.3. Ovalbumin (OVA) Sensitization and Inhalation

As per the modified protocol previously described [16, 17], OVA (500 *μ*g/mL) in PBS was mixed with equal volumes of 10%(w/v) aluminum potassium sulfate (alum; Sigma) in distilled water, then incubated for 60 minutes at room temperature after adjustment to pH 6.5 using 10 N NaOH, and centrifuged at 750 × g for 5 minutes. The OVA/alum pellet was resuspended to the original volume in distilled water. All mice were immunized on two different days (21 days and 7 days before inhalational exposure) by intraperitoneal (i.p.) injections of 0.2 mL alum-precipitated antigen containing 100 *μ*g of OVA (Sigma-Aldrich Korea, South Korea) bound to 4 mg of aluminum hydroxide (Sigma-Aldrich Korea. South Korea) in PBS. Seven days after the second sensitization, mice were exposed to aerosolized OVA for 30 minutes/day, 3 days/week for 12 weeks (at a flow rate of 250 L/minutes, 2.5% OVA in normal saline) and intratracheally injected with 250 *μ*g of OVA (on day 8) on the back of the tongue. PT, CR and their mixture (200, 400 mg/kg) and cyclosporin A (CsA, 20 mg/kg) in solution form were orally administered 3 times a week during the last 8 weeks. One day after the last OVA exposures, airway hyperresponsiveness was determined and samples (blood, bronchoalveolar lavage fluid, lung cells, spleen, peripheral blood mononuclear cells and serum) were collected for further analyses. Blood leukocyte and differential counts were determined by automatic blood count analysis.

### 2.4. Determination of Airway Hyperresponsiveness (AHR)

Airway hyperresponsiveness in mice was estimated using a previously described method with modifications [17, 18]. A Buxco system (Biosystem XA; Buxco Electronics Inc, Troy, Conn, USA) was used to evaluate the extent of airway constriction in different groups of mice following the protocol described previously.

Enhanced pause (Penh) is equal to Pause × PEF/PIF, where Pause = (Te − Tr)/Tr (PIF, peak inspiratory flow; PEF: peak expiratory flow; Te, expiratory time; Tr, relaxation time). In this experiment, mice were aerosolized with OVA for 30 minutes/day, 3 days/week for 12 weeks. At 24 hours after the final inhalation, the mice were given aerosolized normal saline, followed by 3.125, 6.25, 12.5, 25, 50 mg/mL methacholine (Sigma) serially. Airway reactivity was then monitored for 30 minutes. Differences of Penh value between groups were evaluated using a Student's *t*-test.

### 2.5. Bronchoalveolar Lavage Fluid (BALF)

Immediately following assessment of AHR, mice were sacrificed with an i.p. injection of sodium pentoparbitone (100 mg/kg). The trachea was cannulated and BAL fluid were obtained by washing the airway lumina. Briefly, cells in the lungs were recovered by flushing 1 mL of BAL fluid (1 mM EDTA, 10% FBS, PBS) into the lungs via the trachea. Total cell counts were determined and 100 *μ*l of fluid was cytospun onto glass slides using a Cytospin centrifuge (Cellspin, Hanil,, South Korea) (400 g for 4 minutes). Differential cell counts were performed after staining with a Diff-Quik Stain Set (Baxter Healthcare Corp., Miami, Florida, USA). The supernatant of BALF was stored at −25°C for Determination of cytokine levels.

### 2.6. Digestion of Pulmonary Tissue and Cell Preparation

Single cell suspensions from lung tissues and BALF were isolated by mechanical disruption in RPMI 1640 medium supplemented with 2 mM L-glutamine, 100 U/mL penicillin, 100 *μ*g/mL streptomycin, 50 *μ*M 2-mercaptoethanol, 20 mM HEPES, and 2% heat-inactivated fetal bovine serum (FBS, GIBCO, Grand Island, NY, USA). Briefly, the lungs were removed from thoracic cavity. After mincing using sterile scalpels, the tissue was incubated in PBS containing 1 mg/mL collgenase IV and 2 mg/mL dispase for 40 minutes at 37°C in a sterile polypropylene tube. After incubation, lung tissue was vigorously pipetted up and down to further dissolve remaining tissue clumps and then filtered using a 70 *μ*m cell-strainer (Falcon, Le Pont de Claix, France). The total number of cells was counted manually using a hemocytometer chamber (Fisher). Between 2 and 4 × 10^3^ cells were spun onto glass slides (Cytospin centrifuge,Cellspin, Hanil, South Korea) (400 g for 4 minutes). Differential counts were done according to standard morphologic criteria. 

 An amount of 10 IU sodium-heparin dissolved in 1 × D-PBS (with Ca2+ and Mg2+) were used per milliliter of blood from mice. Peripheral blood mononuclear cells (PBMCs) were isolated by centrifugation on Ficoll (density 1.077). They were washed 2 × with D-PBS (with Ca2+ and Mg2+) and used for FACS analysis.

### 2.7. Antibodies and Flow Cytometric Analysis

All antibodies (such as CD3, CCR3, Gr-1, CD11b) to study cell surface markers for different types of leukocytes including T cells (CD3), eosinophil (CCR3), granulocytes (Gr-1) for flow cytometric analysis were purchased from Becton Dickinson (BD) PharMingen (San Diego, Calif, USA). Cells from lung tissues and BALF were stained with the indicated antibodies in staining buffer (PBS containing 1% FBS and 0.01% NaN3) for 10 minutes on ice, and analyzed by two color flow cytometry on a FACSCalibur using CellQuest software (BD Biosciences, Mountain View, Calif, USA).

### 2.8. Quantitative Real-Time PCR

To study the antiasthmatic effects of PT, CR and their combinational prescription on cytokine gene expression from lung tissue, quantitative real-time PCR was performed after quantitative normalization for each gene by densitometry using *β*-actin gene expression. Briefly, total cellular RNA was extracted from the lung by the phenol-chloroform based method (RNAsol^B^: Tel-Test Co. Ltd, USA) according to the manufacturer's instructions. cDNA was synthesized from 3 *μ*g of total RNA using a ReverTraAce-a-cDNA Synthesis kit (Toyobo Co., Ltd. Osaka, Japan) according to the manufacturer's instructions. Real-Time quantitative PCR was performed using the Applied Biosystems 7500 Fast Real-Time PCR system (Applied Biosystems, USA) with the following primers. 

 The primer sequences are as follows: mouse IL-5, 5′-AACCCTTACTGAACTCAGATTGTTAG-3′ and 5′-TAAGTCAGTTTAAATGCTTAGGG-3′ IL-13, 5′-ATGCCCAACAAAGCAGAGAC-3′ and 5′-TGAGAGAACCAGGGAGCTGT-3′*β*-actin, 5′-TGGAATCCTGTGGTCCATGAAAC-3′ and 5′-GTCACAGTCAGCTGTATAGGG-3′. 

 Proinflammatory cytokine gene expression was analyzed with SYBR Green PCR Mastermix (ABI) and a final concentration of 200 nM primers, using *β*-actin as the internal standard. The following PCR parameters were used: 2 minutes at 50°C, 10 minutes 94°C, then 40 cycles of 1 minute at 94°C, and 1 minutes at 60°C. The amount of SYBR Green was measured at the end of each cycle. The cycle number at which the emission intensity of the sample rose above baseline was referred to as the (relative quantitative) RQ and was proportional to the target concentration. Real-Time PCR was performed in duplicate and analyzed by a Applied Biosystems 7500 Fast Real-Time PCR system manual (threshold: 0.05, baseline: 6–15 cycles). To generate standard curves for Proinflammatory cytokine and *β*-actin, serially diluted cDNA (1/1–1/16) was prepared and Real-Time PCR was performed as above. RQ evaluation by RT-PCR was determined and expressed for various samples.

### 2.9. Enzyme-Linked Immunosorbent Assay (ELISA)

Interleukin (IL-4, IL-5, IL-13, eotaxin etc.) and IFN-*γ* production from BALF and the serum of the indicated mice (*n* = 5) was measured by ELISA according to the manufacturer's instructions with a monoclonal antibody-based mouse interleukin ELISA kit (R&D system). OVA-specific IL-4 and IFN-*γ* production from spleen cells were suspended in RPMI 1640 medium supplemented with 2 mM L-glutamine, and 5% fetal bovine serum. The spleen cells were then cultured for 48 hours at a concentration of 1 × 10^5^ cells/well in 96-well culture plates (Corning Inc, Cambridge, Mass, USA) with or without 1 *μ*g/mL of OVA in a humidified atmosphere of 5% CO2 in air at 37°C. The culture supernatants were collected and were assayed for IFN-*γ* and IL-4 antibodies induced by OVA using ELISA. All data represent the mean and standard deviation from at least three separate experiments and were compared using a Student's *t*-test.

### 2.10. Immunohistochemistry (Hematoxyline-Eosin and Masson Trichrome Staining)

Balb/c mice were injected, inhaled and sprayed with OVA for 12 weeks (three times a week) to induce asthma induction. The experimental groups were treated with different concentrations of PT, CR and their combinational prescription for the later 8 weeks (3 times/week). At the end of the experiment, the lungs were removed and analyzed histologically using a modified protocol previously described [19].

### 2.11. Preparative LC Analysis

The water extract was separated by preparative LC (Waters Associates, Milford, Mass, USA) using a ACE 10 C18 column (250 × 30 mm), a model LKB VWM absorbance detector at 214 nm, and a model LKB 2221 integrator. The gradient was solvent B (0 ~ 3: 100% in water), solvent A (3 ~ 20: 0 ~ 100% acetonitrile), and solvent A (20 ~ 30: 100% acetonitrile) for 30 minutes at a flow rate of 30 mL/minutes.

### 2.12. Statistical Analysis

For statistical analysis of data, *P*-values were determined using a one-way analysis of variance (ANOVA) or unpaired Student's *t*-test followed by Dunnett's multiple comparison test (SPSS version 14.0 statistic software). The difference between the normal group and the control group (OVA + vehicle) was clearly distinguished, and for this reason, statistical significance between the normal group and the control group was not shown in the figures and tables to put an emphasis on the statistical differences between the experimental groups and the control group. 

 Results were considered statistically significant if *P* values were <.05 (*), <.01 (**), or <.001 (***).

## 3. Results

### 3.1. Inhibitory Effect of PT, CR, and Their Combinational Prescription on Airway Hyperresponsiveness (AHR)

**Figure 1:**
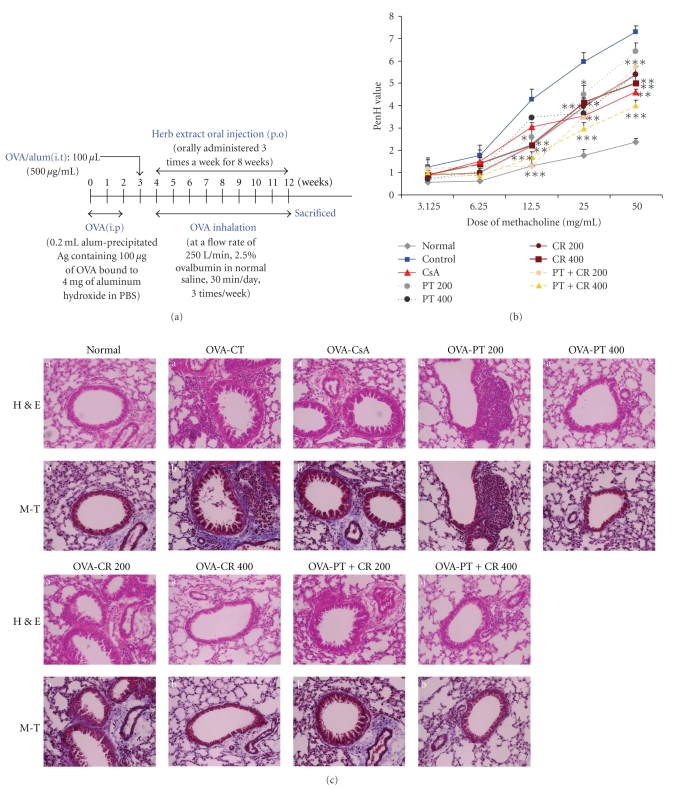
(a), Schematic diagram of methacholine-induced AHR in the sensitization protocol. (b), PenH was measured in a Buxco box, as previously described in materials and methods. *P < .05, **P < .01, ***P < .001 asthma goup (control) versus PT, CR and their combinational prescription treated groups. (c), Effect of PT, CR and their combinational prescription on histology of lung tissue (H&E and M-Tstaining) in lung cells of OVA-induced murine model of asthma. H and E: hematoxylin-eosin stain, M-T: Masson trichrome stain, The results are expressed the mean ± S.E (*N* = 5). Statistically significant value compared with control group data by ANOVA (*P < .05, **P < .01, ***P < .001). N: Normal Balb/c mice, CT: Ovalbumin inhalation (control), OVA + CsA (10 mg/kg), OVA + PT(200, 400 mg/kg), OVA + CR(200, 400 mg/kg), OVA + (PT + CR) (200, 400 mg/kg).

To evaluate the effect of PT, CR and their combinational prescription on airway hyperresponsiveness, total pulmonary airflow obstruction in mice was estimated using a mouse asthma model. Penh was measured using a Buxco system on day 1 after final inhalation and samples were immediately collected. Animals exposed to aerosolized OVA showed increase AHR compared to animals receiving saline only (Figure 1(b)). As shown in Figure 1, relative to animals sensitized with OVA (Control group), PT, CR and their combinational prescription (200, 400 mg/kg) treatment resulted in a significant decrease in methachoine-induced AHR.

### 3.2. Histological Analysis of Lung Sections

We found infiltration of leukocytes in histologic sections of lungs from OVA-exposed control mice, and lung tissue sections from OVA-exposed mice showed airway inflammation and erosion. Eosinophil infiltration was mainly observed in the peribronchial regions of the lung. In contrast, histological sections from herbal extracts-treated mice indicated reduced airway inflammation of lung tissue (Figure 1(c)).

### 3.3. Inhibitory Effect of PT, CR, and Their Combinational Prescription on Airway Eosinophil Accumulation and Influx of Inflammatory Cells into Airways

**Figure 2:**
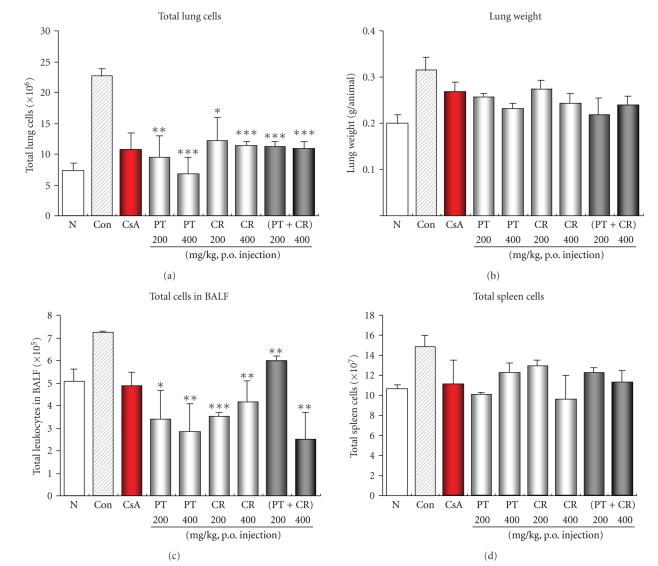
Effects of PT, CR and their combinational prescription on total lung cells, total leucocytes, total lung weight, and total spleen cell. Mice were processed as described in materials and methods, and whole blood was harvested 24 hours after the last OVA challenge. Total inflammatory cell numbers in BALF, lung and spleen were counted, and cell classification was performed on a minimum of 200 cells to classify lymphocytes. The results are expressed the mean ± S.E (*N* = 5). Statistically significant value compared with control group data by ANOVA or unpaired Student's *t*-test (*P < .05, **P < .01, ***P < .001). N: Normal Balb/c mice, CT: Ovalbumin inhalation (control group), CsA: OVA + CsA (10 mg/kg), OVA + PT(200, 400 mg/kg), OVA + CR(200, 400 mg/kg), OVA + (PT + CR) (200, 400 mg/kg).

As shown in Figures 2(a) and 2(c), the total lung cells and total leukocytes in BALF were significantly reduced in PT, CR and their combinational prescription-treated mice compared with control mice, and the number of total spleen cells and lung weight were not significantly reduced in PT, CR and their combinational prescription-treated mice (Figures 2(b)  and 2(d)). 

**Figure 3:**
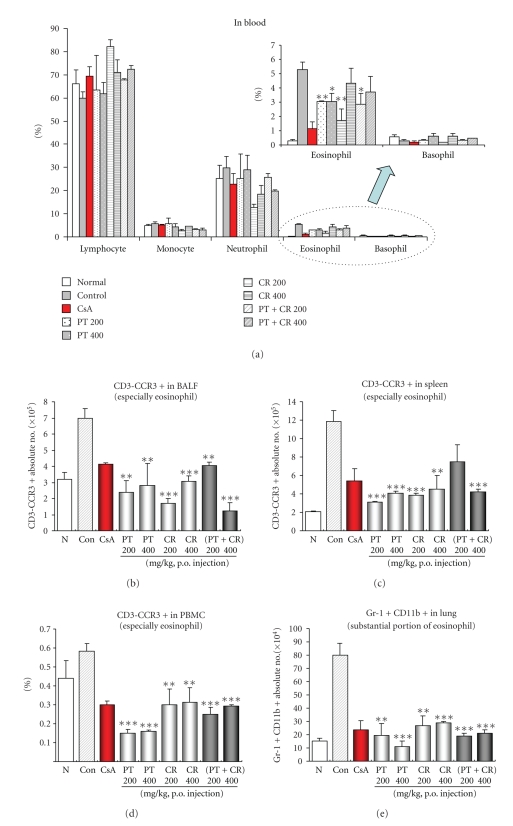
Quantification by means of FACS analysis of CD3-CCR3+ cell subtypes in BALF, spleen, lung, and PBMC. Absolute number of CD3-CCR3+ cell subtypes in BALF, spleen, lung and PBMC were counted (seen materials and methods). The results are expressed the mean ± S.E (*N* = 5). Statistically significant value compared with control group data by ANOVA or unpaired Student's *t*-test (*P < .05, **P < .01, ***P < .001). N: Normal Balb/c mice, CT: Ovalbumin inhalation (control group), CsA: OVA + CsA (10 mg/kg), OVA + PT(200, 400 mg/kg), OVA + CR(200, 400 mg/kg), OVA + (PT + CR) (200, 400 mg/kg).

 Treatment with PT, CR and their combinational prescription resulted in significantly lower numbers of eosinophils (Figure 3(a)), neutrophils (Figure 3(a)), CD3-CCR3+ eosinophils in BALF (Figure 3(b)), spleen (Figure 3(c)) and PBMC (Figure 3(d)) and Gr-1+CD11b+ substantial portion of eosinophils in lung (Figure 3(e)) than control group. Neutrophil number in the bronchoalveolar lavage fluid of mice treated with CR (200 mg/kg) was lower than in immunised mice treated with OVA (Figure 3(a)). In all groups, eosinophil number in bronchoalveolar lavage fluid was lower than in immunised mice treated with OVA. Increased cell infiltration in bronchoalveolar lavage fluid after Ovalbumin challenge was shown in Figure 3(b). However, PT (200 mg/kg) did not affect the absolute number of CD3-CCR3+ eosinophils in spleen (Figure 3(c)).

### 3.4. Inhibition of Cytokines (In Vivo and In Vitro), IgE, and Histamine Production in BAL Fluid and Serum

**Figure 4:**
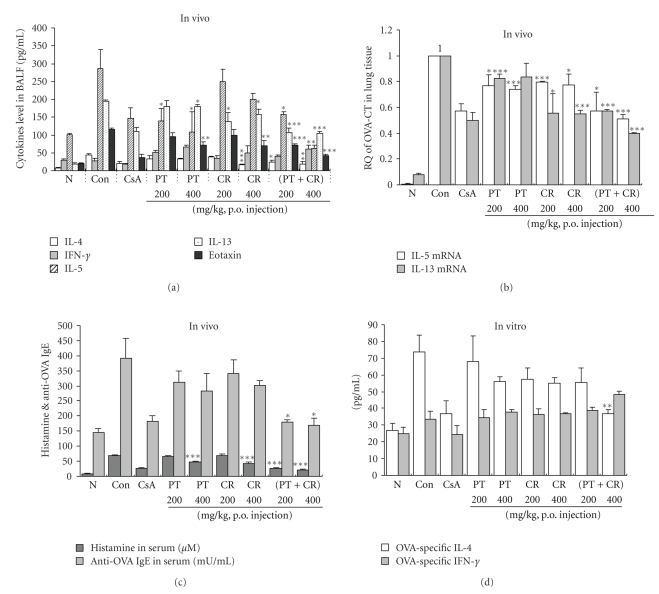
(a), (c), Effect of *Pinellia ternata*, *Citrus reticulata* and their combinational prescription on Th2 cytokines (IL-4, IL-5, IL-13), Th1 cytokine (IFN-*γ*), eotaxin in BALF and IgE, histamine level in serum. (b), Effect of *Pinellia ternata*, *Citrus reticulata* and their combinational prescription on IL-5, IL-13 mRNA gene expression in lung tissue of OVA-induced murine model of asthma (seen materials and methods). (d), Immunomodulatory effects of *Pinellia ternata*, *Citrus reticulata* and their combinational prescription on OVA-specific Th1/Th2 cytokines production in spleen cells (described in materials and methods). The results are expressed the mean ± S.E (*N* = 5). Statistically significant value compared with control group data by ANOVA or unpaired Student's *t*-test (*P < .05, ***P < .001). N: Normal Balb/c mice, CT: Ovalbumin inhalation (control group), CsA: OVA + CsA (10 mg/kg), OVA + PT(200, 400 mg/kg), OVA + CR(200, 400 mg/kg), OVA + (PT + CR) (200, 400 mg/kg).

As shown in Figure 4(a), IL-4, IL-5, IL-13 and eotaxin levels were significantly reduced in PT, CR and their combinational prescription-treated mice. However, they did not affect IFN-*γ* productions in BALF. 

 In our study, serum IgE levels from OVA-induced asthmatic mice were significantly increased compared with control mice (PBS only). PT (400 mg/kg), CR (400 mg/kg) and their combinational prescription (200, 400 mg/kg) had significantly reduced the histamine production (Figure 4(c)). However, only combinational prescription (200, 400 mg/kg) significantly reduced the IgE production (Figure 4(c)). We also measeured IL-4 and IFN-*γ* in the culture supernatants were measured by ELISA and found that combinational prescription (400 mg/kg) significantly inhibited Th2 cytokine (IL-4) production in splenocytes (Figure 4(d)) which was accompanied by a concurrent decrease in Th2 cytokine production in BALF.

### 3.5. Detection of IL-5, IL-13 mRNA in Lung Tissue by SYBR Green Real-Time PCR

As shown in Figure 4(b), the mRNAs for IL-5 and IL-13 were detectable in lung cells treated with PBS only, OVA, and OVA plus PT, CR and their combinational prescription (200, 400 mg/kg) respectively. 

PCR products for IL-5 and IL-13 amplified from lung cell RNA preparations were decreased in the PT, CR and their combinational prescription-treated mice compared with control mice (ova-induced asthma model mice group). This result was accompanied by changes in the eosinophil influx (CD3^−^/CCR3^+^) (Figures 3(b)–3(e)), and BAL cytokines (IL-4, IL-5, and IL-13) production to some degree.

### 3.6. Peak Patterns of PT, CR, and Their Combinational Prescription for Quality Control

**Figure 5:**
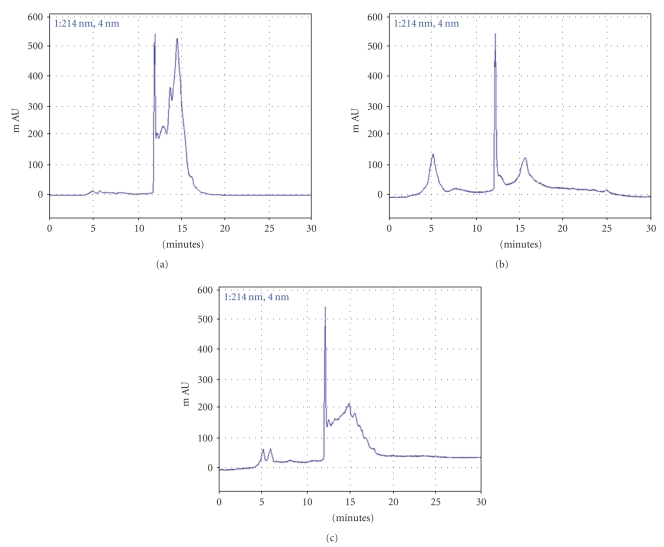
Chromatographic separation of the aqueous extracts of crude (a) PT, (b) CR and (c) their combinational prescription, respectively. Because most of the peaks were eluting within the first 30 minutes, the chromatograms are the displays of the up to 30 minutes.

PT, CR and their combinational prescription were analysed by preparative LC. The chromatogram of them is shown in Figure 5. Peaks of the principal components have not yet been identified in this study.

## 4. Discussion

Therapeutic targets that may be used in the treatment of asthma are numerous. IL-4 blockers either by themselves or in combination with other anti-Th2 cytokines inhibits IgE production and eosinophil infiltration [20]. Because, IL-5 is both a survival and recruitment factor for eosinophils, anti-IL-5 antibody therapy reduces eosinophil adhesion, infiltration and mediator release [21]. Eosinophilia is driven by Th2 cytokines (e.g., IL-4, IL-5, IL-13) produced by Th2 cells. IL-5 is the most critical cytokine mediating increased eosinophil differentiation, maturation, activation, and survival [22]. 

 In this manuscript, we have shown that oral administration of PT, CR and their combinational prescription soften up the phenotype of allergic airway inflammation in BALB/c mice. 

 PT, CR and their combinational prescription prevented the development of AHR (Figure 1(b)), airway eosinophilia (Figure 3), increased Th2 cytokines levels (Figures 4(a) and 4(d)) in BAL fluid and lung inflammation. These results demonstrate that PT, CR and their combinational prescription has profound negative regulatory effects on the development of lung allergic responses in OVA-induced asthma model mice. Moreover, the negative regulatory effects exhibited by them were accompanied by the production of IL-5, IL-13. 

 Asthma produces immune abnormalities in a wide variety of cell populations. Thus, another goal in asthma research would be the evaluation of specific cell subpopulation. 

 In our immunophenotyping by flow cytometry showed a similar pattern as total lymphocyte numbers in BALF and lung. Inhibitory effect of PT, CR and their combinational prescription were observed at 200, 400 mg/kg and hence was used for studying its effect on CD3-CCR3+ eosinophils in BALF, spleen, PBMC, and lung. 

 Eosinophils are attracted, via their CC chemokine receptor 3 (CCR3), in response to chemoattractants such as eotaxin, Rantes, MCP-3, MCP-4 released in the airways of asthmatics [23]. 

 Among chemokines, only CCL11(eotaxin) binds specifically to CCR3 and is the most potent chemokine for movement of these cells [24]. The CCR3 receptor seems to be involved in the activation and degranulation of eosinophils, as well as with the primary migration of the cells, as a number of CCR3 ligands have been reported to induce degranulation of eosinophils [25]. 

 The airway inflammation process that occurs in the development of asthma is characterized by tissue infiltration of eosinophils. 

 Similar to what was seen for cell recruitment (Figure 2), PCR products for IL-5, IL-13 from lung cell RNA preparations were decreased in the PT, CR and their combinational prescription treated group compared with control groups. This results indicate that they significantly affected IL-5, IL-13 mRNAs expression which attract eosinophils and Th2 cells into the airway. 

 Eosinophils are one of the cell types known to express Gr-1 and the eosinophil populations may constitute a substantial portion of the CD11b+Gr-1+ or CD11b+CD49b+Gr-1+ populations [26] that significantly decreased in PT, CR and their combinational prescription treated groups compared to OVA-challenge (Figure 3(e)). 

 Our results show that this is the first study that examines the inhibitory effects of PT, CR and their combinational prescription in lung and BALF on OVA-induced murine model of asthma after inhaled OVA challenge. We observed that inhalation challenge with PT, CR and their combinational prescription administration resulted in a decrease in airway CD11b+ macrophage, Gr-1 granulocytes when compared with that observed after OVA challenge only. Moreover, CD3-CCR3 expression in BALF, spleen, PBMC is downregulated by them (Figures 3(b), 3(c), and 3(d)). 

 Nicholas reported that T helper (Th) type 2 cytokines interleukin (IL)-4 and IL-13 induce the production of specific chemokines through STAT6 signal activated pathways [25]. This chemokine (eotaxin/CCL11) induces migration of Th2 lymphocytes through specific receptors (CCR3). Eotaxin, have also been shown to be potent eosinophil chemoattractants. The continued activation of these cell populations promotes the chronic pathophysiological dysfunction observed during asthma, including mucous production, peribronchial thickening and fibrosis. These pathways might be initiated and maintained through chronic allergen exposure. 

 Therfore, it may be thought that PT, CR and their combinational prescription can reduce Th2 cytokine production and gene expression by inhibition STAT6 activation by above similar mechanism. 

 Pharmaceutical actions, derived from the combination of herbal formula, are called “chemical combination effects”. Kiyohara et al. explained that these complex phenomena are supposed to emerge as a number of different active ingredients interact with each other, acting on different target systems in the body. These types of pharmacological action are called either “pharmacological combination effects” or “pharmaceutical combination effects” [27]. As shown in our results, it would be interesting to identify precisely such therapeutic mechanism affected by them in future studies. Particularly, combinational prescription is more effective in IL-13 and eotaxin production in BALF (Figure 4(a)), but it is not certain that above formula has a chemical combinational effects. 

 In summary, PT, CR and their combinational prescription have deep inhibitory effects on airway inflammation in a murine model of asthma and it was caused by suppression of Th2 cytokines (IL-4, IL-5, IL-13), IgE, eosinophil CCR3 expression in lung. Hence, the results indicated that they could act as a potential immunomodulator by downregulation Th2 cytokines. However, additional studies are needed to characterize the precise mechanism of therapeutic action of them for treatment asthma.
